# Report of Two Surgical Cases

**Published:** 1855-12

**Authors:** W. H. Brown

**Affiliations:** Griffin, Georgia, Professor of Anatomy in the Atlanta Medical College


ARTICLE IV.—*Report of two Surgical Cases.* By W. H. BROWN, M. D., Griffin, Georgia, Professor of Anatomy in the Atlanta Medical College.

CASE 1.—*Hydrocele, with Melanotic Tumor of the Scrotum.* Satisfied fully that we can offer nothing new to our professional brethren by this recital of cases, we perhaps owe an apology for thus presenting them; but, believing as we do, that by carefully arranging and submitting facts, gathered from the fruitful field of practical medicine and surgery, we may oftentimes interest and strengthen each other, familiarize our minds with points of interests, and greatly enhance our usefulness, trust to be excused for thus occupying your columns. Perhaps, too, many of ample experience, skill, and ability, may from similar feelings be induced to disgorge their well-stored note-books of much interesting and valuable matter, particularly to those laboring in the same field, under similar circumstances of climate, institutions, and manners.

In the early part of 1853, a friend and resident of our city, Mr. N., desired we should examine a little son, then about six years of age, in consequence of an unnatural size and appearance of the scrotum.

We found, on examination, a case of hydrocele, and from the history given, pronounced it congenital; advised local bathing with cold water and artificial support of the testicles. We endeavored to quiet his fears as to the termination, and promised a successful operation provided this course failed to effect a cure. Some two or three months later we were called to see him, and found the parts much enlarged and almost black from effusion of blood in the cellular tissue, as the result of accidental contusion, the testicles in natural condition, and serous effusion about the same as when before examined—perhaps somewhat increased; but in addition, and much to our surprise, for a moment, there seemed to be a third testicle, which we directly ascertained to be a well defined, firm tumor, about the size of the testis, and situated on the septum, rather anterior and inferior to those organs.

In a few days the extravisated blood was entirely removed; the temporary soreness and swelling gone; when at the request of the parents we proceeded to remove the tumor. Made a free incision, which, opening into the tunica vaginalis, discharged at once the accumulated serum; brought into view the right testicle, in part, and clearly exhibited the tumor in septum scroti. We carefully removed the tumor, with a portion of the tunica vaginalis. The wound was brought together by the interrupted suture and healed readily by first intention, which confined the little fellow only a few days in doors, and left the parts in a perfectly healthy and normal condition. The hydrocele was radically cured, too, some three years having elapsed without the slightest evidence of its return. The tumor was of the saculated or encysted class, containing a dark, grumous, semi-fluid substance—hence our classification.

CASE 2.—*Gunshot Wound of the Thorax, Pleuritic Effusion, &c.* —About twelve months after excision of tumor—as related above—the same subject, son of Mr. R——, age seven years, was accidently shot with a small pistol. The muzzle of the weapon only a few feet distant, discharged its contents into the left side about two inches below the clavicle. The ball struck the third rib 1½ inch from its articulation with the sterum, and passing in a direction downward and outward, was found resting on the inferior margin of the scapulæ, retained and covered only by the integument. The ball was removed, an anodyne administered and the patient put to bed before I saw him. Proceeding to examine carefully the nature and extent of the injury, we found the small silver probe introduced at the point of entrance—followed the direction mentioned above. There was no evidence of an opening into or communication with the pleural sac, that we could discover—though an opinion to the contrary was expressed by a fellow practioner—on the contrary, the lung seemed to perfom its function uninteruptedly—the inspirations not quite so full as natural, in consequence of soreness of the wound, as expressed by the sufferer. The respiratory murmur was to be heard distinctly over the entire region of the left lung, and no cough or expectoration that would indicate wound of the pleura with abrasion of the lung. At the expiration of some forty-eight hours, ordinary reaction began, and was met by the administration of a full saliue cathartic dose. Shortly after venesection, followed by emollient applications to the region of the wound, were deemed proper.

About the third or fouth day were discharged, by slight cough, sputa, tinged somewhat with darkish or bruised blood—whether from the lung or posterior nares, fauces, &c., it was difficult to ascertain, as there was some cold in the head complained of previous to the accident, and we were not present at such times as they were discharged. Suppuration now began from the wound and went on freely discharging, several times, small spiculæ of bone. No material change was seen to take place until the ninth or tenth day, when increased arterial action, hacking caugh and lancinating pain in the side, indicated plainly pleuritic inflammation. This was met by as active antiphlogistic treatment as was thought safe, and resulted in an apparent subsidence of the inflammation. In a few days, however, another complication presented itself more serious in import than any preceeding—enlargement of the left side, the intercostal spaces protruding even beyond the level of the ribs, rapid breathing, and almost entire suppression of the respiratory murmur in left lung. Before the effects of any remedial agent were perceptable, suppuration continuing free from the wound had established a communication with the cavity, and by a sudden cough was discharged from the wound near half pint of serum. This continued to be discharged by every effort producing contraction of pleural sac atmospheric air entering freely, and being forced out at every expiration, produced the ordinary hissing sound. The discharge soon became sero-purnlent, which was changed gradually to almost pure pus. So the case went on for weeks and months, the external wound frequently closing and requiring to be kept open by the use of tents, &c. During the progress of the case strict and almost exclusive attention was paid to the support of the general system; meeting and combating symptoms as they arose, truly after the expectant mode.

In conducting this case, we feel constrained to say, that no one remedial agent was employed with half the good effect which invariably attended the administration of the *Veratrum Viride.* Without it we verily believe the case would have been lost. Every effort made to substitute other agents resulted in failure—the Veratrum at all times producing the desired effects; controlling the arterial action, promoting gentle perspiration, subduing all pain and uneasiness, (the invariable result of excited circulation,) and bidding defiance to the angry elements, which in their tumultnous onset threatened to overwhelm nature's powers of endurance, made us master of every difficulty and victorious in the end.

Though many are afraid of, and others in doubt as to the virtues and efficiency of the Veratrum, my own experience in the use of it, enables me to express with confidence, the opinion that it is next in importance to opium, the "*Magnum donum deii*," in the treatment of diseases most frequently met with in our Southern latitude. Nor should we soon forget the efforts of him who rescued it from the oblivion, to which it was fast approaching, as a remedy, and placed it in its true light before us.

But to conclude this hastily written article; after a lapse of three or four months adhesion between pleura pulmonalis and cortalis took place, binding down the left lung and causing collapse to a very considerable extent of the ribs of the left side. But a few months have proved sufficient to overcome those adhesions, restore the ribs to their proper position and the lung to a full and perfect performance of its function. The patient is now as active, healthy, and perfect in development as any boy of his age.

